# Effect of *Nigella sativa* oil extracts on inflammatory and oxidative stress markers in Behcet’s disease: A randomized, double-blind, placebo-controlled clinical trial

**Published:** 2020

**Authors:** Shahrzad Amizadeh, Nadereh Rashtchizadeh, Alireza Khabbazi, Amir Ghorbanihaghjo, Ali-Asghar Ebrahimi, Amir-Mansour Vatankhah, Aida Malek Mahdavi, Mohsen Taghizadeh

**Affiliations:** 1 *Connective Tissue Diseases Research Center, Tabriz University of Medical Sciences, Tabriz, Iran*; 2 *Biotechnology Research Center, Tabriz University of Medical Sciences, Tabriz, Iran*; 3 *Drug Applied Research Center, Tabriz University of Medical Sciences, Tabriz, Iran*; 4 *Research Center for Biochemistry and Nutrition in Metabolic Diseases, Kashan University of Medical Sciences, Kashan, Iran*; 5 *Barij Essence Medicinal Plants Research Center, Kashan, Iran*

**Keywords:** Nigella sativa, Behcet’s disease, IL-10, TNF-α, Oxidative stress

## Abstract

**Objective:**

Behcet's disease (BD) is a chronic inflammatory disorder characterized by recurrent oral and genital aphthous ulcers, uveitis and skin lesions. Oxidative stress and inflammation have important role in the pathogenesis of BD. The aim of this study was to assess the effect of *Nigella sativa* (NS) oil administration on malondialdehyde (MDA), total anti-oxidant capacity (TAC), tumor necrosis factor-α (TNF-α), IL-10 and high sensitivity C-reactive protein (hs- CRP) levels in patients with BD.

**Materials and Methods:**

In this randomized, double-blind and placebo-controlled clinical trial, 96 BD patients were randomly assigned to NS or placebo groups. Study groups received 1000 mg/day NS oil and placebo soft gels for 8 weeks. Serum levels of TNF-α, IL-10, hs-CRP, MDA and TAC were measured before and after treatment.

**Results:**

Eighty-nine individuals completed the study. Significant decreases in the serum levels of MDA and increases in the serum levels of TAC were found in the NS group. However, differences in the changes of MDA and TAC in the NS and placebo groups were not significant. Pre- and post-intervention changes of TNF-α, IL-10 and hs-CRP levels in the NS group were non-significant.

**Conclusion:**

NS 1000 mg per day is probably not effective in reducing the inflammatory and oxidative markers in BD.

## Introduction

Behcet's disease (BD) is a chronic inflammatory disorder characterized by recurrent oral and genital aphthous ulcers, uveitis and skin lesions. BD is most commonly observed along the ancient Silk Road (Davatchi et al., 2000 ▶). Despite the unknown pathogenesis of BD, many studies have suggested the role of autoimmunity (de Smet et al., 2000 ▶; Yamamoto et al., 1993 ▶). Environmental factors such as microbial agents and vitamin D deficiency, epigenetics and genetics play a role in inducing abnormal immune responses (Alipour et al., 2017 ▶; Hosseini et al., 2015 ▶; Khabbazi et al., 2019 ▶; Mazzoccoli et al., 2016 ▶). Over-activation of T helper 1 and 2 lymphocytes in parallel with polymorphonuclear leukocytes (PMNs) culminates in liberation of miscellaneous cytokines and overproduction of free radicals, which exert detrimental impact on the cells (Doğan et al., 1999 ▶; Mantaş et al., 1999 ▶; KÖSE et al., 1995 ▶; Mazzoccoli et al., 2016 ▶; Nielsen et al., 1997 ▶; Sharifian et al., 2005 ▶; Takeno et al., 1995 ▶; Turan et al., 2008 ▶). On the other hand, nutrients can control immune responses in living organisms by stimulating or suppressing inflammatory circuits. One of the novel and efficacious anti-inflammatory plants, *Nigella sativa* (NS), known as black seed or black cumin belongs to the Ranunculaceae family and is native to the southern Europe, North Africa and Asia (Schleicher et al., 2000 ▶). NS anti-inflammatory effects are related to suppression of inflammatory cytokines such as interleukin 1β (IL-1β), IL-6 and tumor necrosis factor α (TNF-α), increasing anti-inflammatory cytokines production, like IL-10, stimulation of antioxidant enzymes, and suppression of nuclear factor kappa B (NFκβ) (Budancamanak et al., 2006 ▶; Gheita et al., 2012 ▶; Majdalawieh et al., 2010 ▶; Sayed-Ahmed et al., 2012 ▶). Most of the therapeutic effects emanated from this plant are attributed to an alkaloid called thymoquinone (TQ), which down-regulates a wide variety of the inflammatory cytokines (Budancamanak et al., 2006 ▶; El-Mahmoudy et al., 2002 ▶; Mansour et al., 2004 ▶; Sethi et al., 2008 ▶). TQ in rats with adjuvant-induced rheumatoid arthritis (RA) significantly improved the symptoms and the inflammatory profiles (Tekeoglu et al., 2007 ▶; Umar et al., 2012 ▶). The therapeutic effects of NS in RA were demonstrated (Hadi et al., 2016 ▶). 

Medicinal plants are commonly used in traditional Iranian medicine to treat rheumatic diseases. The immunomodulatory and anti-inflammatory effects of NS make it a good candidate for BD treatment. It is, therefore, important to examine the effect of NS on oxidative stress factors, inflammatory and anti-inflammatory cytokines in patients with BD. To the best of our knowledge, no study was conducted on the effect of NS on oxidative stress factors and various cytokines in BD. This randomized double-blind controlled (RCT) study was conducted to assess the effect of NS oil administration on malondialdehyde (MDA), total anti-oxidant capacity (TAC), TNF-α, IL-10 and high sensitivity C-reactive protein (hs- CRP) levels in patients with BD.

## Materials and Methods

**Study design**

A double-blind placebo-controlled RCT was carried out at Tabriz University of Medical Sciences (TUOMS) from April 2015 until February 2017 and approved by the Ethics Committee of TUOMS. This survey was registered on the website of the Iranian Registry of Clinical Trial (IRCT) (http://www.irct.ir/) with IRCTID: IRCT 201511086975N5. 

**Subjects**

**Figure 1:**
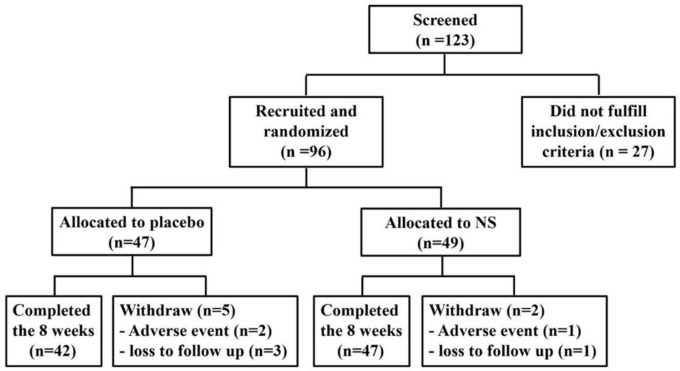
Recruitment and enrollment of the study participants and outcomes

We screened 123 patients with BD and 96 patients were recruited and randomly allocated to the NS (n=49) or placebo (n=47) groups. However, 47 of the NS group and 42 of the placebo group completed the study (Figure 1).

We performed randomization using RandList software 1.2 (http://www.randomisation.eu). BD was diagnosed based on the international criteria of Behcet’s disease (ICBD) (Davatchi et al., 2014 ▶). All the participants were recruited from a BD cohort in the Connective Tissue Diseases Research Center clinic of Tabriz University of Medical Sciences (TUOMS) and they signed an informed consent. The inclusion criteria were (1) age older than 16 years old; (2) no change in medications 2 months prior to the start of study; and (3) no intake of antioxidant supplements from one month before the study. The exclusion criteria were (1) pregnancy and lactation; (2) taking contraceptive pills; (3) hormone replacement therapy; (4) endocrine disorders including thyroid dysfunction and Cushing's syndrome; (5) severe obesity (BMI≥40); (6) chronic diseases such as diabetes mellitus, malignancy or renal dysfunction; (7) any outstanding change in social habits (diet, physical activity and the lifestyle); and (8) being a smoker or a passive smoker.

**Assessments**

The NS and placebo groups received soft gels containing 1000 mg NS oil (Barij Essence, Kashan, Iran) or 1000 mg placebo , respectively, per day 30 minutes before lunch for 8 weeks. Routine therapies continued. From patients, 10 ml blood was taken before and 8 weeks after the commencement of the study. Samples were immediately centrifuged and serum samples were stored at -70°C until biochemical analyses. Serum levels of TNF-α and IL-10 were measured by the enzyme-linked immunosorbent assay (ELISA) kits (IBL, Germany, Ref No_TNF-α_. BE55181 and Ref No_IL-10_. BE53101). hs-CRP was checked by using Monobind ELISA kit (Monobind, USA, Ref No. 1331624). All of these factors were evaluated by the ELISA plate reader (STATFAX-2100, Multi-detection Multi Plate Reader, USA). Finally, TNF-α and IL-10 absorption was determined by a Sicilian spectrophotometer (UK) at 450 nm. hs-CRP’s absorption was assessed by Alcyon 300 autoanalyzer. Based on the instructions provided by the manufacturers of the kits, plates were read at 450 nm (with a reference wave length of 630 nm). MDA measurement as an oxidative product was done based on the reaction with thiobarbituric acid (TBA), extraction with normal butanol, spectrophotometer absorption measurements at 532 nm, and comparison of the absorptions with the standard curve (Bilici et al., 2001 ▶). In this measurement, Cecil spectrophotometer (UK) was utilized. To measure TAC, Randox, UK Kit (Cat No. NX2332) was used (Miller et al., 1993 ▶). Then, the initial light absorption of the cuvettes was measured at 600 nm by using Alcyon 300 auto analyzer at 37°C in the air. The final light absorption was determined three minutes after adding 200 μl of the substrate to each cuvette.

**Statistical analysis**

Statistical analysis of the data was performed by SPSS software version 24.0. At first, both of the groups were examined for sex and age. Then, the Kolmogorov-Smirnov test was utilized to determine the normal or skewed distribution of the data in both groups. According to the results of this test, MDA, TAC and age were analyzed by parametric methods. While TNF-α, hs-CRP and IL-10 were evaluated by non-parametric statistical methods.

Independent-T and Mann-Whitney U tests were used to compare the homogeneity of the groups before the intervention. The male/female ratio in the study groups was compared by Chi-Square test. Then, pre- and post-intervention data were compared in both groups by using Paired Sample-T test (as a parametric method) and Wilcoxon Test (as a non-parametric equivalent). Finally, the correlations between the quantitative data (∆MDA, ∆TAC, ∆TNF-α, ∆IL-10, and ∆hs-CRP) were analyzed by using the Pearson parametric and Spearman non-parametric tests. A p value less than 0.05 was considered statistically significant.

## Results

**Subjects**

Ninety-six patients suffering from BD were enrolled in the study. Eighty-nine individuals completed the study (Figure 1). Demographic and clinical characteristics and medications of studied groups at the baseline, are presented in Table 1. The differences were not significant (p>0.05).

**Table 1:** Demographic and clinical characteristics and medications of studied groups at the baseline

**Variables**	***Nigella sativa*** ** group** **(N=47)**	**Placebo group** **(N=42)**	**p-value**
**Age, mean (±SD), years old**	39.8±10.2	38.3±10.2	NS*
**Male : Female**	31:16	24:18	NS†
**Disease duration, months **	10 (min 4, max 35, mode 5)**	9 (min 1, max 44, mode 4)**	NS**
**Clinical Manifestations**			
Oral aphthous ulcer (%) Genital ulcer (%) Skin lesions (%) Uveitis (%) Pathergy (%) Arthritis (%) Vascular involvement (%) CNS involvement (%) GI involvement (%)	36 (97.3) 21 (56.8) 14 (37.8) 16 (43.2) 12 (32.4) 7 (18.9) 5 (13.5) 2 (5.4) 0 (0.0)	33 (97.1) 17 (50.0) 16 (47.1) 16 (47.1) 13 (38.2) 6 (17.6) 5 (14.7) 3 (8.8) 1 (2.9)	NS NS NS NS NS NS NS NS NS
**Medications**			
Prednisolone (%) Colchicine (%) Azathioprine (%) Interferon-ɑ (%) Methotrexate (%) Cyclosporine (%) NSAID (%) Mycophenolate Mofetile (%) Infliximab (%) Sulfasalazine (%) Cyclophosphamide (%)	27 (73.0) 13 (35.1) 10 (27.0) 6 (16.2) 5 (13.5) 1 (2.7) 1 (2.7) 1 (2.7) 2 (5.4) 2 (5.4) 1 (2.7)	28 (82.3) 10 (29.4) 12 (35.3) 2 (5.9) 7 (20.6) 1 (2.9) 4 (11.8) 2 (5.9) 3 (8.8) 0 (0.0) 0 (0.0)	NS NS NS NS NS NS NS NS NS NS NS

NS=Non-significant; CNS=Central nervous system involvement; NSAID=Nonsteroidal anti-inflammatory drug; GI=Gastrointestinal; SD=Standard deviation; *Performed using Independent-Sample T Test; †Performed using Chi-Square Test; ** 2 Independent Samples, Mann- Whitney U Test

**Biochemical analysis**

**Figure 2:**
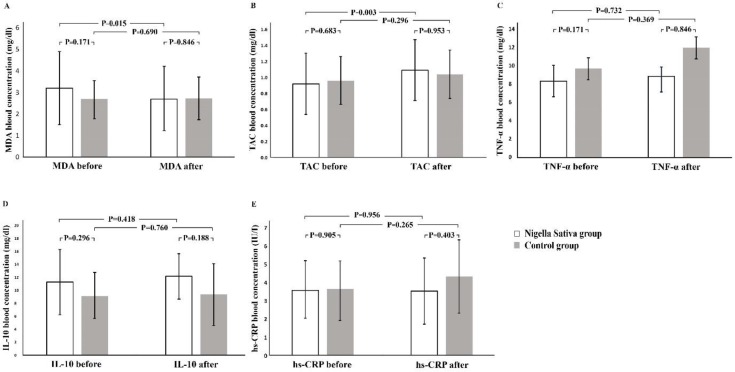
Cytokine profile and stress oxidative markers levels in the *Nigella sativa* and placebo groups

According to Kolmogorov-Smirnov test, IL-10, TNF-α and hs-CRP had skewed distribution and were evaluated by the nonparametric tests. However, MDA and TAC were analyzed by parametric equivalents. Thereafter, the pre- and post-intervention data were compared separately in both groups. This analysis showed that the TAC level increased significantly and the MDA level decreased significantly pre and post-intervention in the NS group (Figure 2A and B). On the other hand, changes in the TAC and MDA levels in the placebo group were not significant (Figure 2A and B).

MDA= Malondialdehyde; TAC= total anti-oxidant capacity; TNF-α= tumor necrosis factor-α; IL-10= interleukin-10; and hs-CRP= high sensitive C-reactive protein.

Pre and post-intervention levels of hs-CRP, TNF-α and IL-10 levels in the NS and placebo groups were not significantly different (Figure 2C, D and E). Thereafter, the pre- and post-interventional changes in values were compared separately in studied groups. We compared the pure alteration of the values between the NS and placebo groups (∆= after minus before). Minus and plus signs besides the calculated digits disclosed decreased and increased levels after intervention. This comparison showed no significant differences in the ∆TAC, ∆MDA, ∆hs-CRP, ∆IL-10 and ∆TNF-α, between the studied groups (Tables 2 and 3).

**Table 2:** Correlations between the changes in MDA, and TAC levels after 8 weeks of treatment with *Nigella sativa* and placebo

**Parameters**	***Nigella sativa*** ** group**				**Placebo group**			
	**∆MDA**		**∆TAC**		**∆MDA**		**∆TAC**	
	r	p value	r	p value	r	p value	r	p value
**∆MDA**	-	-	- 0.71	0.001**	-	-	-0.83	0.001**
**∆TAC**	- 0.71	0.001**	-	-	-0.83	0.001**	-	-

∆MDA=MDA after 8 weeks of placebo treatment minus MDA at the baseline; ∆TAC=TAC after 8 weeks of placebo treatment minus TAC at the baseline; r=Correlation coefficient; **Pearson’s Test was used to determine correlations between ∆MDA and ∆TAC.

**Table 3:** Correlations between the changes in TNF-α, IL-10, hs-CRP levels after 8 weeks of treatment with *Nigella sativa* and placebo

**Parameter**	***Nigella sativa*** ** group**						**Placebo group**					
	**∆TNF-α**		**∆IL-10**		**∆hs-CRP**		**∆TNF-α**		**∆IL-10**		**∆hs-CRP**	
	r	p value	r	p value	r	p value	r	p value	r	p value	r	p value
**∆TNF-α**	-	-	0.06	0.66 *	0.18	0.2 *	-	-	0.12	0.44*	0.08	0.59*
**∆IL-10**	0.06	0.66 *	-	-	0.16	0.26*	0.12	0.44*	-	-	-0.47	0.002*
**∆hs-CRP**	0.18	0.2 *	0.16	0.26*	-	-	- 0.08	0.59*	0.47	0.002*	-	-

*Spearman’s rho was used to determine correlations between ∆ TNF-α, ∆ IL-10 and ∆ hs-CRP; Δ TNF-α=TNF-α (after 8 weeks of placebo treatment) minus TNF-α (baseline); Δ IL10=IL-10 (after 8 weeks of placebo treatment) minus IL-10 (baseline); ∆ hs-CRP=hs-CRP (after 8 weeks of placebo treatment) minus hs-CRP (baseline); r=Correlation coefficient

## Discussion

The present study showed that 8 weeks of treatment with NS 1000 mg per day compared with placebo, did not significantly change MDA, TAC, hs-CRP, IL-10 or TNF-a levels.

TNF-α is a very important inflammatory cytokine that dramatically increases in chronic inflammatory disorders (Yoruk et al., 2010 ▶). IL-10 is one of the important anti-inflammatory cytokines and its amplification *in vivo* suppresses inflammatory reactions and modulates the immune responses (Ahmed and Hassanein, 2013 ▶). hs-CRP is a pentameric protein synthesized in hepatocytes in response to several inflammatory agents (Ebru et al., 2008 ▶). hs-CRP amplifies the process of the phagocytosis and activation of the complement system. Its reduction discloses a relatively silent circumstance without grave inflammatory reactions in the body. Hadi et al. (2016) ▶, similar to our study, did not find significant changes in the amount of TNF-α using NS in RA patients. However, they found a dramatic increase of IL-10 in patients treated with NS (Hadi et al., 2016 ▶). Mahdavi et al. found that NS in combination with a low-calorie diet can significantly reduce TNF-α and hs-CRP levels (Mahdavi et al., 2016 ▶). 

Many studies showed the role of oxidative stress in BD pathogenesis and its correlation with BD manifestations. Najim et al. (2007) ▶ in a study on 76 BD patients, showed increased levels of serum MDA. Harzallah et al. (2008) ▶ measured plasma MDA levels, reduced glutathione (GSH)/oxidized glutathione (GSSG) ratio, erythrocyte superoxide dismutase (SOD), catalase and glutathione peroxidase in 40 patients with BD. They found higher MDA and catalase and lower GSH/GSSG ratio and SOD levels in BD patients compared with healthy controls. MDA is a toxic oxidant that is produced as a hazardous by-product during lipid peroxidation in the body and amplifies during extensive oxidative stress (Nielsen et al., 1997 ▶). The free radicals produced during the biochemical reactions in the body, may invade the biomolecules including lipids with at least 3 double bonds (Gawel et al., 2004 ▶). Distortion of the above-mentioned lipids by free radicals, leads to MDA production. TAC assesses the antioxidant status of biological samples and can evaluate the antioxidant response to free radicals produced in a particular disease (Sharifian et al., 2005 ▶). Antioxidant agents can neutralize a wide variety of free radicals in the body by increasing the TAC. This study did not show the antioxidant effects of NS in BD patients. Contrary to our findings, in a study conducted on women with RA, MDA was significantly lower in the NS-treated group (Yoruk et al., 2010 ▶). Hadi et al. also found a significant reduction in MDA and increase in TAC levels in RA patients treated with NS extract (Hadi et al., 2016 ▶). 

To the best of our knowledge, this study was the first clinical trial which evaluated the effects of NS on MDA, TAC, TNF-α, IL-10 and high sensitivity C-reactive protein (hs- CRP) levels in patients with BD. 

However, our study had some limitations. Short duration of intervention, small sample size, using low dose of NS (1000 mg per day) and treating BD patients with immunomodulatory medications during the study which may blur the effects of NS, were the main concerns. Designing another study with a higher number of patients, longer duration of treatment with NS and using different doses of NS may solve these problems.

NS 1000 mg per day is probably not effective in reducing the inflammatory and oxidative markers in BD. 
